# Valorization of Waste from Lavender Distillation Through Optimized Encapsulation Processes

**DOI:** 10.3390/foods14152684

**Published:** 2025-07-30

**Authors:** Nikoletta Solomakou, Dimitrios Fotiou, Efthymia Tsachouridou, Athanasia M. Goula

**Affiliations:** Department of Food Science and Technology, School of Agriculture, Forestry and Natural Environment, Aristotle University, 541 24 Thessaloniki, Greece; nsolomak@agro.auth.gr (N.S.); dmfotiou@agro.auth.gr (D.F.); etsachou@agro.auth.gr (E.T.)

**Keywords:** co-crystallization, encapsulation efficiency, hydrodistillation, ionic gelation, phenolic compounds

## Abstract

This study evaluated and compared two encapsulation techniques—co-crystallization and ionic gelation—for stabilizing bioactive components derived from lavender distillation residues. Utilizing aqueous ethanol extraction (solid residues) and concentration (liquid residues), phenolic-rich extracts were incorporated into encapsulation matrices and processed under controlled conditions. Comprehensive characterization included encapsulation efficiency (Ef), antioxidant activity (AA), moisture content, hygroscopicity, dissolution time, bulk density, and color parameters (L*, a*, b*). Co-crystallization outperformed ionic gelation across most criteria, achieving significantly higher Ef (>150%) and superior functional properties such as lower moisture content (<0.5%), negative hygroscopicity (−6%), and faster dissolution (<60 s). These features suggested enhanced physicochemical stability and suitability for applications requiring long shelf life and rapid solubility. In contrast, extruded beads exhibited high moisture levels (94.0–95.4%) but allowed better control over morphological features. The work introduced a mild-processing approach applied innovatively to the valorization of lavender distillation waste through structurally stable phenolic delivery systems. By systematically benchmarking two distinct encapsulation strategies under equivalent formulation conditions, this study advanced current understanding in bioactive microencapsulation and offers new tools for developing functional ingredients from aromatic plant by-products.

## 1. Introduction

The valorization of waste materials from industrial processes has become an increasingly important area of research and development, particularly in the context of sustainability and circular economy principles. One such waste stream that presents significant potential for value-added applications is the by-products generated from lavender distillation process. Lavender (*Lavandula* spp.) is widely cultivated for its essentials oils, which are extensively used in the food, pharmaceutical, and cosmetic industries. However, the distillation process results in a substantial amount of residual biomass, which, if not properly managed, can contribute to environmental pollution and resource wastage. Therefore, developing efficient strategies to utilize these residues is crucial for enhancing sustainability and economic viability [1,2].

Distillation remains the most commonly employed method for extracting essential oil from lavender, with steam distillation and hydro-distillation being the predominant techniques. The efficiency of these methods is influenced by multiple parameters, including the type of distillation equipment, operational parameters, and the quality of the lavender feedstock. The distillation process results in several by-products, collectively referred to as lavender distillation waste. Among these, hydrolats (hydrosols) and leachates are notable liquid by-products with significant potential for revalorization. Hydrosols, produced through the condensation of steam used in the extraction of essential oils, contain a portion of volatile aromatic compounds, thereby rendering them significant components in cosmetic formulations and aromatherapy products. Their application provides an opportunity to minimize waste while enhancing the economic return from lavender cultivation. Leachates, on the other hand, comprise aqueous mixtures rich in high-molecular-weight, water-soluble components that are released from the plant matrix during thermal processing but are not captured in the distillation procedure. These leachates frequently encompass bioactive phenolic compounds that exhibit antioxidant, antimicrobial, and therapeutic characteristics, rendering them appropriate for functional uses in nutraceuticals, pharmaceuticals, and food preservation.

Furthermore, the solid waste generated from the distillation of lavender plants is identified as a valuable source of phenolic acids (such as p-coumaric, neochlorogenic, caffeic, and rosmarinic acid) and flavonoids (including quercetin-3-β-glucoside, quercetin, and mircetine), which exhibit antioxidant and antimicrobial properties [3]. Few studies focused on the extraction processes of phenolic compounds from lavender solid waste [2,4,5].

However, although polyphenols derived from lavender by-products exhibit promising bioactivity, their practical application is often constrained by their susceptibility to degradation during food processing, transportation, storage, and gastrointestinal transit. Environmental factors such as light, heat, oxygen, and pH fluctuations can significantly reduce the stability and efficacy of these components, whether they are applied in food, pharmaceutical, or cosmetic formulations [6,7]. To counter these limitations, the development of protective delivery systems has emerged as a key focus in improving the efficacy and shelf life of phenolic-rich extracts.

Among the various strategies for the valorization of lavender distillation waste, the encapsulation of extracts obtained from these by-products has emerged as a particularly promising and effective approach. Encapsulation technologies enable the stabilization, protection, and controlled release of bioactive components, thereby enhancing their functional properties and extending their applicability across diverse industrial sectors. By employing advanced encapsulation methods such as co-crystallization and ionic gelation, it is possible to optimize the recovery and functional integration of valuable bioactive constituents derived from lavender distillation waste. These techniques not only improve the solubility and bioavailability of value-added compounds but also extend their shelf life, making them more viable for commercial applications.

Co-crystallization has emerged as a novel encapsulation technique, lauded for its cost-effectiveness, operational simplicity, and its ability to significantly improve the physicochemical characteristics of encapsulated bioactives. By forming irregular, porous sucrose matrices through controlled crystallization, this method can effectively entrap phenolic components, thereby enhancing their solubility, flowability, dispersibility, and stability while also mitigating undesirable sensory attributes such as bitterness [8,9]. Despite its potential, the application of co-crystallization for encapsulating botanical extracts, especially those derived from lavender waste, remains underexplored. Nonetheless, recent studies show promising outcomes. For instance, Sarabandi et al. [10] demonstrated the successful co-crystallization of anthocyanin-rich sour tea extract, while González-Reza et al. [11] applied this method to phenolic-rich cactus pear waste, achieving improved antioxidant retention. Tzatsi and Goula [12] reported an optimal co-crystallization efficiency of 96.3% using unused chokeberries, whereas Chezanoglou et al. [13] achieved encapsulation efficiencies ranging from 33.68 to 113.02% with pomegranate peel extract. Earlier research has also shown the method’s versatility in stabilizing a variety of natural extracts and oils, pointing to its broad applicability in functional food and nutraceutical development [14,15,16,17].

An increasingly efficient approach to preserving the functional and health-enhancing characteristics of plant bioactive compounds involves their incorporation into biopolymer-based delivery systems, which can notably enhance both stability and bioavailability. Among available biopolymers, alginate stands out as one of the most widely used due to its natural source, biocompatibility, and ability to form hydrogels under mild conditions. The ionic gelation technique relies on the interaction between alginate and multivalent cations—commonly calcium ions—forming cross-linked gel structures without the need for harsh chemicals, thus maintaining the safety of the final product for food and pharmaceutical use [18,19]. The gelation process typically involves the dropwise addition of alginate solution into a calcium chloride bath, triggering the formation of hydrogel beads. This mechanism is well-explained by the “egg-box” model, which illustrates how calcium ions coordinate with the guluronate blocks of alginate chains to form stable junction zones [12]. Recent studies have demonstrated the efficacy of ionic gelation in encapsulating phenolic-rich extracts. For instance, Silveira et al. [20] encapsulated pomegranate polyphenols using pectin-based hydrogel beads formed by ionic gelation, achieving encapsulation efficiencies up to 101%. Similarly, Tzatsi and Goula [12] reported encapsulation efficiencies between 8.94 and 93.87% for extracts of unused chokeberries, whereas Vakarelova et al. [21] explored the microencapsulation of astaxanthin with various gelling polymers through ionic gelation, resulting in improved stability and bioaccessibility. Several other studies further underscore the versatility and effectiveness of ionic gelation [22,23,24,25,26].

To date, research on the encapsulation of phenolic components derived from lavender distillation residues remains limited, particularly with respect to advanced techniques such as ionic gelation or co-crystallization. Therefore, the present work aims to optimize the use of lavender leachates and solid by-products by developing suitable encapsulation strategies tailored to enhance the stability and functionality of their bioactive compounds. For each method, experimental design methodologies were employed to investigate the influence of key process parameters on encapsulation efficiency. The physicochemical characteristics—such as moisture content, bulk density, hygroscopicity, color, antioxidant potential—were thoroughly assessed. The resulting powder forms of the encapsulated extracts may serve as value-added functional ingredients for the development of enriched food products, supporting the integration of bioactive plant metabolites into the food chain and promoting circular bioeconomy approaches.

## 2. Materials and Methods

### 2.1. Plant Material

Lavender (*Lavandula stoechas*) used in this study was sourced from local cultivators in Rodolivos (Rodolivos IKE, Serres, Greece). The plant material, exhibiting a moisture content of approximately 18 ± 1% (wet basis), was harvested during the full-bloom stage in June 2022 to ensure a high concentration of bioactive compounds (~18 mg GAE/g). Entire aerial parts of the plant—including stems, leaves, and flowers—were collected and, subsequently, they were manually separated from the remaining plant material and stored in airtight bags at ambient temperature until further processing.

### 2.2. Hydrodistillation

Hydrodistillation was carried out using a Clevenger-type apparatus (LG-11125-102, Wilmad-LabGlass, Warminster, PA, USA). A 30 g portion of lavender plant material was placed into a 1 L round-bottom flask containing 600 mL of distilled water. The distillation was conducted for 105 min using an electric heating mantle (Model 655, Nahita Ltd., London, UK), yielding approximately 1.5 mL of essential oil. Prolonged distillation beyond this point resulted in negligible increases in oil recovery. The distillation process yielded the following fractions:

- Essential oil (EO);
- Solid waste (SW): residual plant biomass remaining in the flask post-distillation;
- Liquid waste or leachate (LW): wastewater in the glass flask;
- Hydrolate or floral water (Hydro): the aqueous distillate composed of condensed steam enriched with water-soluble aromatic constituents.

### 2.3. Preparation of Phenolic Extracts from Distillation By-Products

Phenolic extracts were derived from both the solid (SW) and liquid (LW) by-products obtained after the hydrodistillation of lavender. The LW was concentrated under reduced pressure (150 mbar) at 40 °C using a rotary evaporator (Model R 114, Buchi Laboratoriums-Technik, Büchi, Flawil, Switzerland).

In parallel, the solid waste (SW), composed primarily of spent lavender biomass, was dried in a forced-air convention oven at 40 °C for 24 h, aiming to reduce water activity, thereby minimizing the rate of undesirable degradation reactions and inhibiting microbial growth. A drying temperature of 40 °C was selected to avoid the thermal degradation of heat-sensitive components. Once dried, the material was stored in airtight bags at ambient temperature until extraction.

In our previous work [2], both ultrasound-assisted extraction (UAE) and microwave-assisted extraction (MAE) methods were evaluated for their effectiveness in recovering polyphenol from lavender distillation solid waste. Among the two, MAE proved to be more efficient, delivering superior extraction yields. Under optimized MAE conditions—800 W microwave power, a solvent-to-solid ratio of 30 mL/g, and 34% aqueous ethanol for 4 min—the extraction yield reached 14.75 ± 0.35 mg gallic acid equivalents (GAE)/g dry weight (d.w.). Therefore, MAE was selected for the current study to extract phenolic components from lavender distillation solid waste.

Following extraction, the resulting phenolic extracts were concentrated according to the total solids content (°Brix) required by the encapsulation experimental designs. The concentrated extracts were then stored in airtight containers at −20 °C until further use.

### 2.4. Encapsulation

#### 2.4.1. Encapsulation by Ionic Gelation

**Figure 1:**
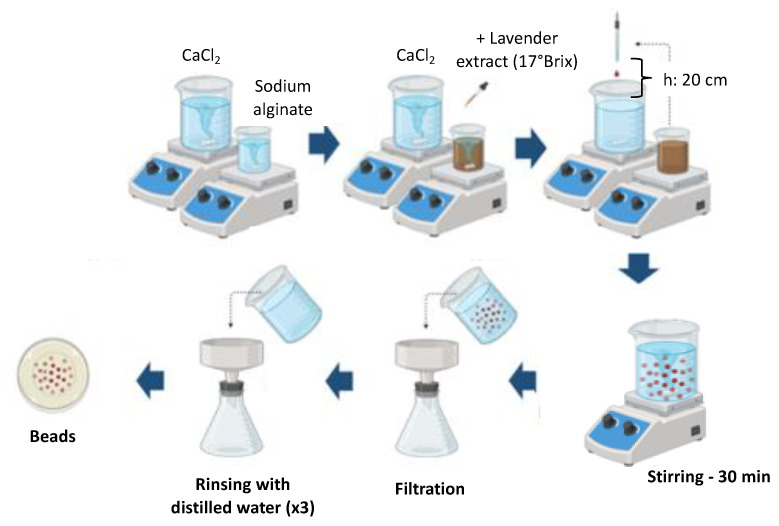
Schematic diagram of the ionic gelation method.

A sodium alginate solution with a concentration ranged from 2 to 3% w/w (Calg) was prepared and mixed with lavender by-product extracts concentrated to 17 °Brix. The concentration of extract in the mixture (Ce) varied between 1 and 15% w/w. Based on the experimental design (Table 1), nine different formulations were evaluated, following a modified version of the method described by Tzatsi and Goula [12]. The resulting mixture was thoroughly homogenized at 500 rpm for 30 min and subsequently dispensed dropwise into a 2.5% (w/v) calcium chloride solution using a 5 mL pipette with a tip diameter of 1.43 ± 0.03 mm, from fixed height of 20 cm to ensure consistent bead formation (Figure 1). The formed beads were allowed to remain in the crosslinking solution for 30 min to complete gelation. Afterward, they were collected by filtration and rinsed three times with distilled water.

#### 2.4.2. Encapsulation by Co-Crystallization

**Figure 2:**
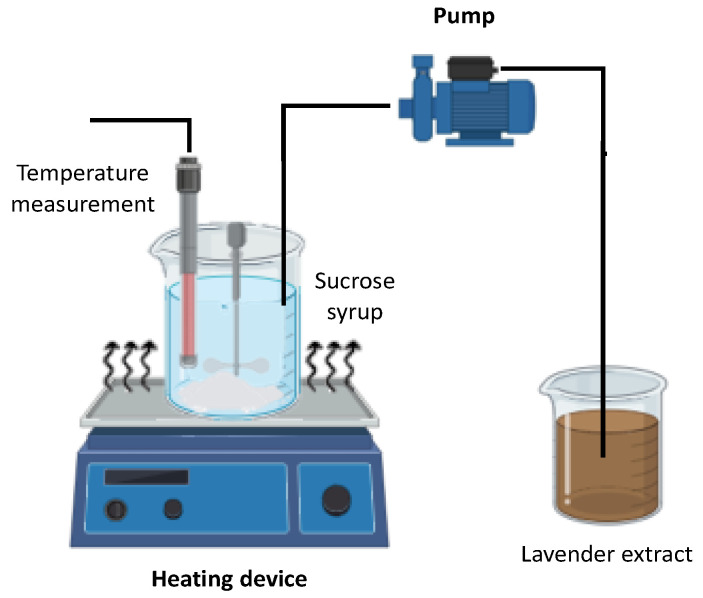
Schematic diagram of the co-crystallization method.

Sucrose (75 g) was dissolved in 12.5 mL of distilled water and the solution was continuously stirred at 500 rpm using a vertical agitator (IKA Eurostar 40 digital, Staufen, Germany) in a cylindrical stainless-steel container (8 cm in height, 0.8 cm wall thickness, and 6.8 cm bottom diameter) placed on a heating plate, as illustrated in Figure 2 and described by Chezanoglou et al. [13]. The mixture was heated on a hot plate with magnetic stirring until it reached the target temperature of 140 °C. Stirring during heating facilitates uniform crystallization [27], while progressive evaporation at elevated temperature increased the concentration of the sucrose syrup from 70–75 to a 90–95 °Brix [17]. The onset of crystallization was indicated by the appearance of slight turbidity due to spontaneous nucleation under supersaturated conditions [8].

Upon reaching the desired temperature (~140 °C), heating was discontinued, and the stirring speed was increased to 700 rpm. Subsequently, lavender distillation waste extract, at ambient temperature and with varying total soluble solids content (35–70 °Brix), was rapidly added to the syrup at different dry extract-to-sucrose ratios ranging from 0.1 to 0.7 g/g (Table 1). The mixture was immediately transferred to a water bath maintained at 25 °C and stirred continuously until the temperature decreased below 60 °C, stabilizing around 45 °C. The entire co-crystallization process, from initial heating to the final product formation, was completed within 19–21 min. The resulting powder was collected, transferred to a sealed glass container, and stored in a desiccator for 24 h.

### 2.5. Determination of Encapsulation Efficiency

Encapsulation efficiency (Ef, %) is defined as the proportion of total phenolic components successfully entrapped within the encapsulated product. For both ionic gelation and co-crystallization encapsulation techniques, efficiency was calculated according to the following formula:

Ef = (L_c_/L_o_) × 100 (1)

where L_c_ is the amount of phenolic compounds in the final encapsulated product and L_o_ is the initial amount of phenolic compounds in the extract of the waste (SW, LW) prior to encapsulation.

To determine the phenolic content in the extruded beads, 6 g of chopped alginate beads were extracted with 20 mL of MeOH. The extraction was performed in an ultrasonic water bath Sonorex Digiplus (Bandelin, Berlin, Germany) (temperature: 20 °C, time: 30 min, intensity: 50%). The resulting extract was collected by vacuum filtration, concentrated using rotary evaporator, and the total phenolic content was quantified using Folin–Ciocalteu method, as described by Tzatsi and Goula [12].

In the case of co-crystallized powders, 0.2 mL of the sample was dispersed in 10 mL of distilled water, stirred thoroughly, and filtered through a 0.45 μm membrane filter. The clear filtrate was then subjected to total phenolic content determination using the same analytical method. Specifically, 0.5 mL of Folin–Ciocalteu reagent was added to the filtrate and the mixture was vigorously shaken. After 5 min, 5 mL of a 5% Na_2_CO_3_ solution was added under continuous mixing. The final volume was adjusted to 25 mL using distilled water, thoroughly mixed, and then left to stand for 90 min. The absorbance of the resulting solution was measured at 750 nm against an appropriate blank.

### 2.6. Characterization of the Encapsulated Products

#### 2.6.1. Moisture Content

The moisture content (%, wet basis) was determined by drying encapsulated samples in an oven at 105 °C until a constant weight was achieved. After drying, the sample was allowed to cool in a desiccator at room temperature.

#### 2.6.2. Solubility-Wetting Time

Solubility was assessed by dissolving 1 g of powder in 25 mL of distilled water at room temperature, under continuous stirring at 900 rpm using a 2 mm × 7 mm magnetic stirring bar in a 150 mL glass beaker. The method involved measuring the time required for the powder to completely sink beneath the water surface, referred to as the wetting time, which serves as an indicator of solubility performance [12,13].

#### 2.6.3. Color

An automatic Minolta colorimeter (CR-400, Konica-Minolta Sensing, Inc., Tokyo, Japan) was employed to evaluate the color characteristics of the encapsulated phenolic extracts. The samples were uniformly spread in 5 cm-diameter Petri dishes to a thickness of 0.5 cm. Color measurements were conducted using the CIE-L*a*b* color space (CIE-Lab), which quantifies lightness (L*), red–green (a*), and yellow–blue (b*) components, providing an objective assessment of color attributes.

#### 2.6.4. Bulk Density

A 2 g sample of the powder was accurately weighted and transferred into a 50 mL graduated cylinder without compaction. The bulk density (g/mL) was determined as the ratio of the powder mass to the volume it occupied within the cylinder, providing an estimate of the powder’s packing behavior under gravity.

#### 2.6.5. Hygroscopicity

Approximately 1 g of powder was evenly distributed on 9 cm diameter Petri dishes to maximize the surface area exposed to air. The samples were then placed in a desiccator maintained at 23 °C and 76% relative humidity, established using a nitric acid (HNO_3_) solution. Moisture sorption kinetics were assessed by recording weight changes at 10 min intervals. The moisture uptake was expressed as the increase in mass per gram of powder solids, reflecting the powder’s hygroscopic behavior under controlled humidity conditions [12].

#### 2.6.6. Beads Size

To characterize the beads, their diameter was measured using a caliper on a sample of 8–10 randomly selected beads.

#### 2.6.7. Morphology

The morphology of the encapsulated products was examined using an XTX-5C Step stereo microscope (Hinotek, Ningbo, China) at magnifications of 20×–80×, under an accelerating voltage of 10 kV.

#### 2.6.8. Antioxidant Activity

The antioxidant activity of the samples was assessed based on their free radical scavenging capacity using the 1,1-diphenyl-2-picrylhydrayl (DPPH) reagent. Specifically, 1 g of the encapsulated extract was dissolved in 10 mL of distilled water. An aliquot of 100 μL from the resulting solution was then mixed with 3.9 mL DPPH ethanolic solution. The mixture was incubated in the dark for 30 min, after which the absorbance was measured at 517 nm until the reaction plateaued. The radical scavenging activity, expressed as percentage inhibition (I, %), was calculated using the following equation:

(2) $$I\;(\%)\;=\;(\frac{A_{b}{-A}_{s}}{A_{b}})\;\times \;100\%$$

where A_b_ is the absorbance of the blank, and A_s_ is the absorbance of the sample.

This analysis was performed individually for all encapsulated samples produced using both co-crystallization and ionic gelation methods, and for each type of lavender distillation residue (SW, LW) evaluated in the study.

### 2.7. Statistical Analysis

To evaluate the significance of individual effects and their interactions, an analysis of variance (ANOVA) was conducted. Differences were considered statistically significant at p-value < 0.05. Experimental data were processed using Minitab statistical software (Release 15, MINITAB Inc., Lock Haven, PA, USA) to identify optimal experimental conditions for maximizing encapsulation efficiency.

All experiments and analytical measurements were performed in triplicate (n = 3), and results are expressed as mean ± standard deviation.

## 3. Results and Discussion

### 3.1. Encapsulation Efficiency

#### 3.1.1. Ionic Gelation

In the present study, the efficiency of the ionic gelation technique was systematically investigated for the encapsulation of extracts obtained from solid residues of lavender distillation. The primary focus was placed on the influence of two critical independent variables: sodium alginate concentration (Calg, %), serving as the gelling matrix-forming agent, and extract concentration (Ce, %), representing the bioactive core material.

**Figure 3:**
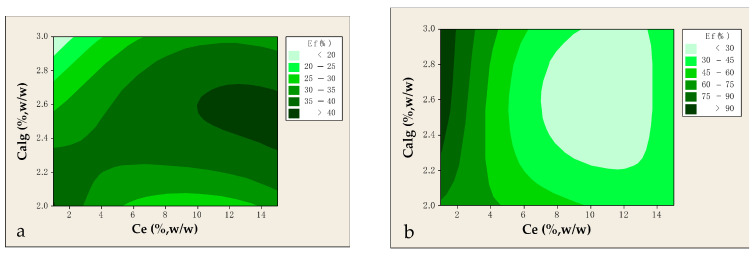
Effect of sodium alginate concentration (Calg, % w/w) and extract concentration (Ce, % w/w) on encapsulation efficiency (Ef, %) of beads produced by ionic gelation using (**a**) solid and (**b**) liquid by-products from lavender distillation.

According to the experimental results (Figure 3a), the encapsulation efficiency (Ef) ranged between 15.83 and 41.08%, with a maximum value of 41%, observed at Calg and Ce concentrations of 2.5 and 15%, respectively. Throughout all encapsulation experiments, the CaCl_2_ concentration was maintained constant at 2.5% (w/v). This concentration was selected based on established literature data. Specifically, Tzatsi and Goula [12] reported that increasing CaCl_2_ concentration from 2.0 to 2.5% led to a notable enhancement in Ef, which was attributed to increased crosslinking density and improved gel rigidity. This is consistent with the known behavior of Ca^2+^ ions, which promote the formation of more compact and mechanically stable alginate beads due to intensified ionic interactions with the guluronic acid blocks. However, a further increase in CaCl_2_ concentration beyond 2.5 (w/v) has been shown to negatively affect the thermodynamic stability of alginate hydrogels. As Zhang et al. [28] demonstrated, exceeding the calcium-binding capacity of alginate leads to site saturation, thereby impeding efficient additional crosslinking and compromising the structural integrity of the hydrogel network.

In this work, both the reduction in Calg to 2.0% and its increase to 3.0% resulted in diminished encapsulation efficiency, as observed in the contour plots derived from experimental data (Figure 3a). This trend is likely attributable to the suboptimal rheological characteristics of the alginate solution at both concentration extremes. At lower concentrations, the reduced viscosity may be insufficient to effectively entrap solutes during bead formation. In contrast, higher alginate concentrations are associated with elevated viscosity, which can hinder the uniform dispersion of the core material and compromise the formation of mechanically stable and structurally homogeneous beads. This observation is consistent with the well-established correlation between alginate concentration and solution viscosity, which directly affects the gelling and encapsulation behavior of the biopolymer. Moreover, no bead formation was observed at alginate concentrations below 2% (w/v), likely due to the inadequate availability of carboxyl functional groups required for the formation of discrete spherical beads [26,28]. Moreover, the Ce appeared to exert a positive influence on Ef, as a gradual increase in extract concentration from 1 to 15% led to a steady enhancement in entrapment yield. This phenomenon may be explained by the reduced diffusivity of the extract in the aqueous medium during the fixed curing time, as reported by Toprakçı et al. [29], who encapsulated a sour cherry biowaste in calcium alginate beads.

In a subsequent experimental series, the influence of Calg and Ce on Ef was further investigated using the extract derived from the liquid phase of lavender distillation. As illustrated in Figure 3b, increasing Calg from 2.0 to 3.0% led to a modest enhancement in Ef, presumably due to improved gel strength and structural integrity of the hydrogel matrix. However, the beneficial effect was limited, likely constrained by either excessive matrix viscosity or saturation of encapsulation capacity [12,30]. In stark contrast, increasing Ce from 1% to 15% induced a pronounced decline in Ef, from approximately 90% to 40%, indicative of a destabilizing influence of the liquid extract on bead formation and stability. This inverse relationship may be ascribed to physicochemical perturbations such as increased osmotic pressure, ionic strength imbalances, or competitive interactions between phenolic constituents and Ca^2+^ ions, which may hinder effective crosslinking. Mehta et al. [31] have emphasized the detrimental effects of high core load concentrations on encapsulation systems, noting that excessive core material may compromise the integrity of the polymeric barrier, promote leakage, and reduce encapsulation yield and functional performance. Figure 3b further elucidated the interactive effects of Calg and Ce. Ef consistently decreased when Ce exceeded 6–8%, regardless of Calg concentration, suggesting that phenolic overloading disrupts matrix cohesion, likely through saturation of binding sites or disruption of gel microarchitecture. Conversely, increased Calg concentrations mitigated the decline in Ef at low Ce levels, reinforcing the stabilizing role of alginate; however, this effect was insufficient to counteract the destabilizing influence of elevated Ce levels. These results underscore the criticality of matrix-to-core ratio in designing efficient encapsulation systems. Furthermore, studies by Toprakçı et al. [29] and Liu et al. [32] corroborate these findings, emphasizing the delicate balance required between alginate and extract concentration to achieve maximal encapsulation efficiency.

Under optimal conditions (Calg = 3.0%, Ce = 1.0%), the experimental Ef reached 102.22 ± 0.90%, denoting near-complete encapsulation. The regression model exhibited a high degree of fit to the experimental data, with R^2^ of 91.15%, although the overall level of statistical significance was marginal (*p* = 0.082), suggesting that only specific terms may have contributed meaningfully to the observed variability. In particular, the concentration of the liquid residue (Ce) was found to be statistically significant (*p* = 0.017, F = 23.71), confirming its predominant role in influencing encapsulation efficiency. In contrast, the Calg did not exert a statistically significant effect (*p* = 0.611). These findings reinforce the observation that Ce acts as the primary regulatory factor in this encapsulation system, while Calg plays a supportive role without independently affecting the response in a statistically significant manner.

#### 3.1.2. Co-Crystallization

Co-crystallization represents a particularly promising encapsulation technique, exploiting the ability of sucrose to form stable crystalline lattices during the transition from solution to solid state, thereby simultaneously entrapping bioactive components. In the present study, the influence of two key variables—the solids concentration of the extract (Xs, °Brix) and the extract-to-sucrose ratio (E/S, g/g)—on the encapsulation efficiency (Ef, %) of phenolic compounds derived from lavender solid waste extract was investigated.

**Figure 4:**
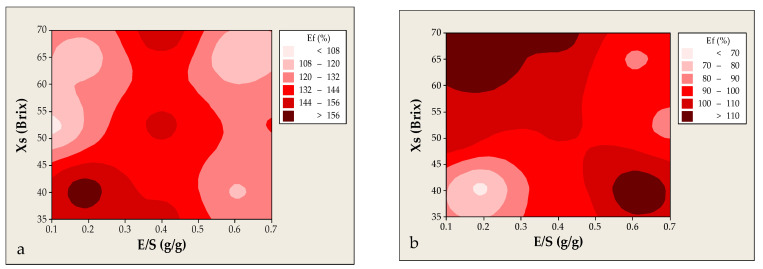
Effect of solids concentration of extract (Xs, °Brix) and dry extract-to-sucrose ratio (E/S, g/g) on encapsulation efficiency (Ef, %) of co-crystallized powders produced using (**a**) solid and (**b**) liquid by-products from lavender distillation.

As illustrated in Figure 4a, Ef exhibited a nonlinear response to changes in Xs. Based on experimental data, the highest encapsulation yields (>150%) were observed at the extremes of the investigated concentration range, namely at approximately 35 and 65–70 °Brix. In contrast, a marked decline (~90%) was recorded at intermediate concentrations around 52.5 °Brix. The observed nonlinear behavior of Ef in relation to Xs during sucrose co-crystallization can be attributed to variations in the fluidity and supersaturation levels of the crystallizing medium. At lower Xs values (e.g., ~35 °Brix), the medium exhibits lower viscosity, facilitating enhanced diffusion and integration of the extract into the forming crystalline matrix. This is supported by studies indicating that decreased viscosity in sucrose solutions promotes better diffusion of solutes, thereby improving entrapment efficiency [33]. Conversely, at higher Xs values (e.g., 65–70 °Brix), the increased concentration leads to a more supersaturated and viscous solution, which favors the stabilization of the crystalline structure. Such conditions enhance the formation of a robust crystalline lattice capable of effectively encapsulating bioactive components [14,33]. Intermediate concentrations (around 52.5 °Brix) may correspond to transitional states between semi-fluid and supersaturated phases. In these states, the medium’s properties are not optimal for either efficient diffusion or stable crystal formation, leading to reduced encapsulation efficiency. This phenomenon has been observed in studies where intermediate sucrose concentrations resulted in less effective co-crystallization processes.

The inverse relationship between the extract-to-sucrose ratio (E/S) and Ef observed in our study aligns with findings reported in the recent international literature. As the E/S ratio increases, the crystalline matrix may become saturated with bioactive components, potentially disrupting the orderly development of sucrose crystals and introducing hygroscopic substances that hinder matrix stabilization. For instance, comparable findings were documented by Chezanoglou et al. [13], who examined co-crystallization of pomegranate peel extract with sucrose. Their findings demonstrated that encapsulation efficiency was maximized at the lower and upper extremes of extract concentration (Xs), while significantly reduced yields were recorded at intermediate concentrations. This phenomenon was ascribed to changes in the viscosity and physicochemical stability of the crystallizing medium, which likely compromised the uniform development of the sucrose crystal network. Similarly, Ali et al. [34] observed that a greater volume of crude Poniol fruit extract in the co-crystallization process contributed to higher entrapment yield.

It should be noted that encapsulation efficiency values exceeding 100% may be attributed to two main factors. First, the co-crystallized product tends to absorb moisture from the environment, and this hygroscopic behavior may introduce minor inaccuracies in the mass determination of the final product, leading to slightly overestimated encapsulation efficiencies. Moreover, possible interactions between the extract constituents and reagents may result in the formation of reaction products that are detectable by the Folin–Ciocalteu method, thereby influencing the absorbance measurements obtained via spectrophotometry—phenomena also reported by other researchers [13]. Additionally, this effect may also stem from the thermal degradation of phenolic components at elevated co-crystallization temperatures, which can lead to the generation of degradation products that still react with the Folin–Ciocalteu reagent, thus contributing to higher absorbance values [35].

In the case of co-crystallization of the liquid waste of lavender distillation, the results differed significantly from those observed during the encapsulation of extracts derived from the solid waste of the same distillation process. Specifically, in the liquid residue, an increase in total solids concentration was accompanied by a progressive rise in Ef, with peak values exceeding 110% observed at 70 °Brix and E/S ratio of 0.4 g/g. This outcome suggests that increasing the solid concentration of an extract may enhance either the availability of phenolic components for entrapment or the stabilization of the crystalline matrix—likely through increased viscosity and reduced water mobility [12,13,34,36]. This positive effect may also be attributed to improved interactions between the concentrated residue at higher Brix levels and sucrose, which could facilitate its incorporation into the developing crystalline network. Figure 4b depicts the combined effect of the two examined factors (Xs and E/S) on Ef, as observed from experimental data, highlighting two distinct optimization regions. High Ef values (>110%) are observed both at high Xs (≥60 °Brix) with moderate E/S (0.10–0.50 g/g) and at lower Xs (≈35–40 °Brix) combined with high E/S (0.55–0.70 g/g). Among the investigated parameters, only Xs had a statistically significant effect (*p* = 0.047), suggesting a direct correlation with Ef, in contrast with the E/S ratio, which had no significant individual impact (*p* = 0.527).

### 3.2. Encapsulated Products Properties

#### 3.2.1. Ionic Gelation

**Figure 5:**
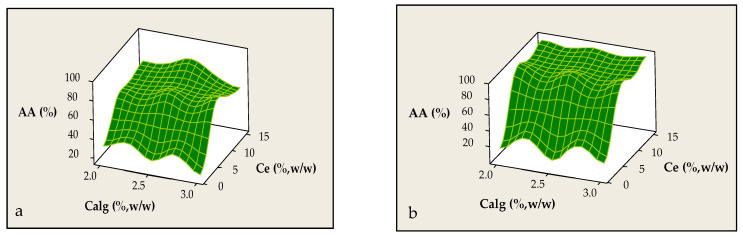
Effect of sodium alginate concentration (Calg, % w/w) and extract concentration (Ce, % w/w) on antioxidant activity (AA, %) of beads produced by ionic gelation using (**a**) solid and (**b**) liquid by-products from lavender distillation.

The preservation of antioxidant activity during encapsulation is critical for the functionality and application of micro-structured systems. In this study, the effects of Calg and Ce of the solid waste extract on antioxidant activity (AA, %) of beads were evaluated. As shown in Figure 5a, AA ranged from ~18 to 80% with a peak (~80%) demonstrated at Calg of 2.5% (*p* = 0.669) but slightly declined at 3%, likely due to gel microstructure changes that hinder phenolic retention. Ce had a more pronounced, statistically significant effect (*p* = 0.008): increasing Ce from 1 to 7.5% significantly boosted AA (>70%), which then plateaued or slightly decreased at 15%, possibly due to saturation effects or phenolic degradation. Similar trends were reported by Bevan et al. [37] for the *Hibiscus sabdariffa* L. extract encapsulated in calcium alginate beads. Figure 5a confirms that maximum AA values occur at Calg = 2.6–2.8% and Ce ≥ 10%, while values of Ce < 5% consistently result in low AA (<40%) regardless of polymer concentration.

In contrast, during the encapsulation of the liquid waste of lavender distillation, the variable Calg does not appear to significantly affect antioxidant activity (*p* = 0.613), as AA values remain relatively stable (~68–70%) across the investigated range of Calg, indicating no significant effect on phenolic entrapment or protection against oxidative degradation. On the other hand, Ce emerges as a critical factor (*p* = 0.000): increasing Ce from 1 to 15% results in a sharp increase in AA, from approximately 20 to over 90% (Figure 5b). Similar trends were reported by Ismaili et al. [38], where increasing the concentration of lavender essential oil in polylactic acid (PLA) nanofibers significantly enhanced AA. Notably, samples containing extract at 12.5% w/w exhibited the highest effectiveness across all applied assays. This supports a strong correlation between bioactive load and antioxidant capacity, consistent with the findings of the present encapsulation study.

**Figure 6:**
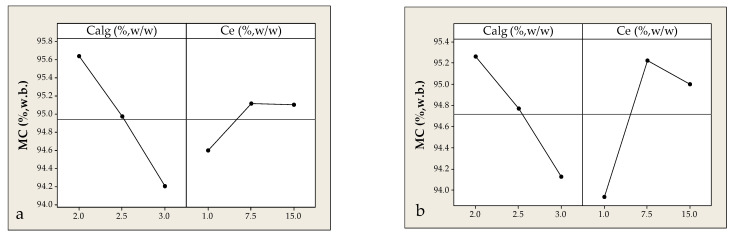
Main effects plot of sodium alginate concentration (Calg, % w/w) and extract concentration (Ce, % w/w) on moisture content (MC, % w.b.) of beads produced by ionic gelation using (**a**) solid and (**b**) liquid by-products.

Moisture content (MC, %) is a critical physicochemical property influencing the stability, rheology, and microbiological safety of encapsulated systems. In alginate beads produced from solid waste extracts, MC increased with Ce up to ~10–12%, after which it stabilized or slightly declined. This may reflect saturation of hydrophilic gel surfaces or reduced extract incorporation at higher Ce levels. Conversely, increasing Calg from 2 to 3% resulted in a steady decrease in MC, likely due to denser gel network formation that limits water retention by reducing hydrophilic cavities. Ce showed a more complex effect: as Ce increased from 1 to 7.5%, MC rose from ~94% to ~95%, likely due to the hygroscopic nature of phenolic components (Figure 6a). However, at 15% Ce, MC slightly declined or plateaued, possibly due to matrix saturation or reduced permeability. Although the overall MC variation was modest (~1%), the observed trends are considered technologically significant.

In case of encapsulation using the liquid by-product from distillation process, MC was similarly affected by Calg. Within the examined Ce range (1.0–7.5%), a comparable increasing trend in MC was observed, while at Ce of 15%, a slight decrease occurred—likely due to matrix saturation and the formation of a denser gel network. Maximum MC was recorded at Ce = 7.5% and Calg = 2%, conditions that combine a medium load of hydrophilic components with a gel micro-structure favorable for moisture retention (Figure 6b). In contrast, the lowest MC (~94%) occurred at high Calg (3%) and low Ce (1%), where structurally cohesive and less hydrophilic beads are likely formed.

Similar observations have been reported in the international literature. Kučuk et al. [39] encapsulated mango peel extract in alginate beads and attributed the increased MC to hygroscopic nature of phenolic due to hydrogen bonding between phenolic groups and water molecules. Likewise, Toprakçi et al. [40] observed that increasing Calg during rosemary phenolic encapsulation reduced moisture levels, attributed to the formation of more compact gels with fewer hydrophilic regions, which facilitated water release. In general, alginate-based gels are highly hydrophilic and can retain up to 90% of their weight in water [41]—a finding confirmed by all experiments in this study, as moisture values consistently exceeded this threshold.

**Figure 7:**
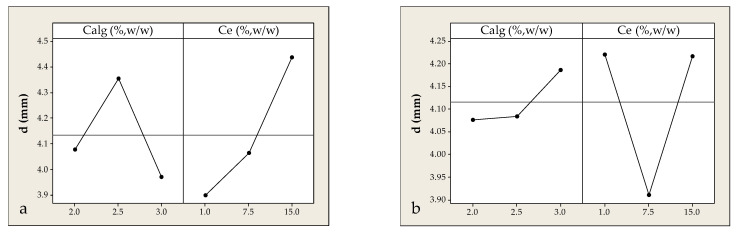
Main effects plot of sodium alginate concentration (Calg, % w/w) and extract concentration (Ce, % w/w) on bead size (d, mm) of beads produced by ionic gelation using (**a**) solid and (**b**) liquid by-products from lavender distillation.

Bead size is a key physicochemical parameter in ionic gelation, affecting mechanical stability and release kinetics. In this work, the effects of Calg (%) and Ce (%) on bead diameter (d, mm) were evaluated. The largest beads (~5.0 mm) formed at Ce ≈ 15% and Calg ≈ 2.5% for experiments conducted in extracts derived from solid distillation by-products, with deviations from these values resulting in a reduced diameter—highlighting the system’s nonlinear behavior. As shown in Figure 7a, both low (2%) and high (3%) Calg concentrations yielded smaller beads, indicating that optimal size requires simultaneous tuning of both investigated parameters. Bead formation in alginate-CaCl_2_ systems also depends on physical parameters such as nozzle diameter, drop height, and surface tension [26]. Generally, higher solution viscosity yields larger droplets [42], while overly fluid solutions at low Calg may fail to retain droplet shape, and highly viscous solutions may limit flow, producing smaller beads. Machado et al. [43] reported similar trends in Spirulina phenolic encapsulation, where increasing Calg from 1 to 2% significantly enlarged bead diameter from 2.64 mm to 3.3 mm, attributed to improved rheology and droplet stability. High Ef (88.97%) and phenolic stability under simulated gastrointestinal conditions were also reported, emphasizing the crucial role of rheological and structural optimization. A positive correlation was also observed between Ce and bead size: increasing Ce from 1 to 15% led to a diameter rise from ~4.0 to ~4.3 mm, likely due to the enhanced solid-phase content and intermolecular interactions during initial droplet formation prior to gelation.

The influence of Calg and Ce on bead diameter was also examined for beads prepared from the liquid distillation residue. As shown in Figure 7b, increasing Calg from 2 to 3% lead to a gradual rise in bead diameter (from ~4.06 to ~4.18 mm), attributed to increased viscosity and the formation of a denser, more cross-linked gel network. In contrast, Ce exhibited a nonlinear effect, with a diameter minimum (~3.9 mm) at Ce ≈ 7.5%, and higher diameters (~4.2 mm) observed at both lower (1%) and higher (15%) Ce levels. This suggests the existence of a critical Ce range in which phenolic or water-soluble components may alter system rheology or interfere with Ca^2+^–alginate interactions, affecting gel formation. Unlike the solid extract system, where maximum diameter occurred at Calg ≈ 2.5%, the largest beads (~4.3–4.4 mm) in the liquid residue system were observed at Calg ≈ 3.0% and Ce ≈ 15%, indicating that higher polymer concentration may offset the disruptive effects of the liquid matrix. Notably, a marked diameter reduction was observed at intermediate Ce (~7.5%) even under high Calg, supporting the hypothesis that specific residue compositions may inhibit proper gelation—possibly via ion competition, delayed network formation, or shrinkage phenomena during gel development. The trends observed here are consistent with findings from other alginate-based systems. Zazzali et al. [44] demonstrated that stronger Ca^2+^–alginate crosslinking and higher extract load led to stable bead structures when using artichoke waste extracts. Machado et al. [43] also found that increasing Calg significantly enlarged bead diameter in Spirulina-based systems due to improved droplet stability. Furthermore, Kuhn et al. [45] showed that phenolic–protein co-encapsulation can interfere with network formation due to ionic interactions or saturation effects.

**Figure 8:**
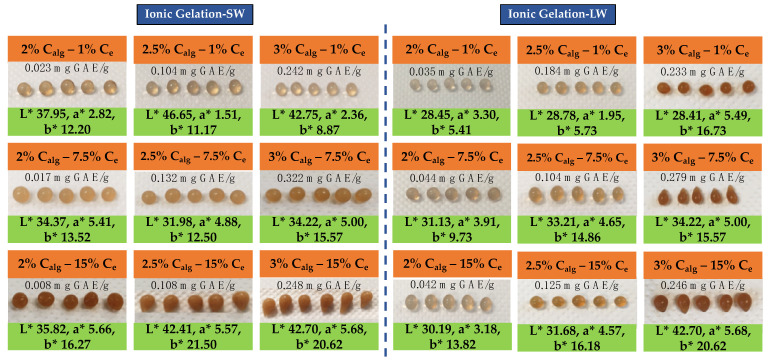
Morphology, color parameters, and phenolic content of encapsulated extracts of lavender distillation solid waste (SW) and liquid waste (LW) by the ionic gelation method.

The color profile of encapsulated alginate beads is a key quality attribute influencing consumer perception. As shown in Figure 8, lightness (L*) increased from ~37 to 41 as Calg ranged from 2 to 2.5%, with a slight decrease at maximum investigated concentration, suggesting that intermediate alginate levels promote the formation of a more homogeneous and translucent gel matrix, enhancing light reflection and diffusion. The effect of Ce on L* parameter was nonlinear: lightness decreased (L*~35) at Ce = 15%, possibly reflecting changes in microstructure. A similar but more nuanced pattern was observed for the a* parameter (red–green axis): Calg had minimal influence, indicating that polymer matrix composition alone does not significantly shift red tones. In contrast, increasing Ce from 1 to 15% markedly elevated a* values from ~2.6 to ~4.9, attributed to higher levels of phenolic and pigment components enhancing red coloration. Regarding the b* parameter (yellow–blue axis), Calg had a limited effect, while Ce had a strong impact, with b* rising from approximately 11 (Ce = 1%) to over 19 (Ce = 15%). This increase may reflect the accumulation of yellowish components or oxidative by-products at high extract concentrations. These observations are consistent with the findings of Kowalonek et al. [46], who reported that alginate films incorporating phenolic-rich oils (e.g., raspberry and blackcurrant seed oils) exhibited notable modifications in color parameters, including increased b* and reduced a*, underscoring the visual impact of natural pigment integration in biopolymer systems.

In this study, the effects of the aforementioned parameters on the color attributes were also evaluated for beads produced of the liquid distillation by-product. In this case as well, the L* value exhibited a mild positive correlation with increasing Calg, possibly due to the formation of a more transparent and homogeneous gel matrix that enhances light reflection. The influence of Ce was more pronounced: L* increased up to Ce ≈ 7.5%, suggesting an optimized distribution of pigment components, while at higher Ce (15%), a decrease in lightness was recorded, as observed in beads produced by the extract of solid distillation residue—potentially due to matrix oversaturation with phenolics or other dark-colored substances. These results highlight Ce as a major parameter in modulating bead lightness, showing a clear tendency for L* to decrease at extreme extract concentrations. In contrast, the effect of Calg on L* was comparatively limited, displaying a slight negative correlation—possibly due to increased light scattering in denser, less transparent gel structures.

#### 3.2.2. Co-Crystallization

When comparing the encapsulation outcome of solid and liquid distillation residues via co-crystallization, distinct differences emerge in their influence on antioxidant activity (AA). In systems derived from solid distillation residues, increasing Xs positively influenced AA (*p* = 0.119). Specifically, AA increased sharply from ~15 to over 80% as Xs rose from 35 to 40 °Brix and remained high (AA > 75%) up to 70 °Brix. This broad stability suggests effective retention of phenolic components, either through incorporation into the developing crystalline network or through stabilizing interactions with sucrose. A slight decline observed near 65 °Brix may be associated with matrix saturation effects or a reduction in molecular mobility, potentially limiting diffusion and entrapment of active compounds.

**Figure 9:**
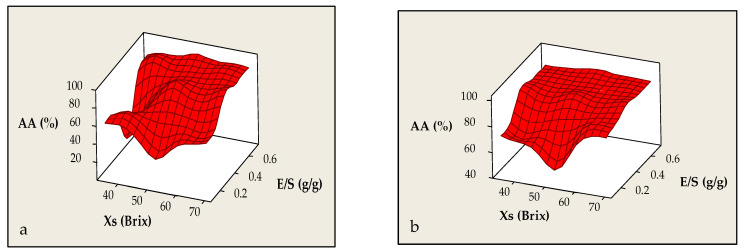
Effect of solids concentration of extract (Xs, °Brix) and dry extract-to-sucrose ratio (E/S, g/g) on antioxidant activity (AA, %) of co-crystallized powders produced using (**a**) solid and (**b**) liquid by-products from lavender distillation.

The E/S ratio also exhibited a strong positive correlation with antioxidant response (*p* = 0.033). Increasing E/S from 0.15 to 0.70 g/g led to a near-linear increase in AA, reaching ~90%, suggesting that higher phenolic load promotes both encapsulation efficiency and final product bioactivity. It is also possible that the presence of elevated extract concentrations slows the crystallization process, allowing more efficient temporal integration of bioactive compounds into the solid matrix. The combined effect of the two investigated parameters (Xs × E/S, *p* = 0.717) is visualized in the contour plot (Figure 9a). The highest AA values (>80%) were observed in zones where Xs exceeded 55 °Brix and E/S ranged from 0.30 to 0.70 g/g. These conditions appear to promote stable integration and preservation of phenolics within the final product, likely through enhanced intermolecular interactions with sucrose. In contrast, at low E/S values (<0.30 g/g), AA dropped below 40%, reflecting insufficient phenolic loading and reduced system functionality. According to Figure 9a, E/S appears to have a reinforcing role, especially under high Xs conditions, while at lower Xs, its influence diminishes—likely due to insufficient matrix density for effective entrapment. This finding underscores that high antioxidant activity cannot be achieved solely by increasing extract concentration; it also requires coordinated adjustment of solution density and E/S ratio. Similar findings have been reported by Ali et al. [34], who achieved 76.38% Ef and 65.10% antioxidant retention through co-crystallization of the Poniol fruit extract. These results reinforce the idea that strategic optimization of both Xs and E/S is critical to the design of micro-structured products with enhanced stability and functionality.

In contrast, the co-crystallized powder derived from the liquid distillation residue of lavender exhibited a different response. AA decreased from ~90 to ~75% as Xs increased from 35 to 40 °Brix (*p* = 0.135), possibly due to transient matrix instability or partial inhibition of the entrapment mechanism—potentially related to changes in viscosity or reduced phenolic availability (Figure 9b). However, from this point onward, AA progressively recovered, reaching >9 5% at 70 °Brix. This recovery is likely attributed to the gradual enhancement of the crystalline network, which facilitates the reestablishment of efficient entrapment. It is important to note that the phenolic composition of the extracts differed substantially between the two waste streams due to extraction method. In the case of solid distillation residues, phenolic compounds were recovered using microwave-assisted extraction in aqueous ethanol solution, a process known to enhance the yield and diversity of phenolic species. Μicrowave extraction of phenolic compounds from solid residue is non-selective and yields phenolic solutions with large amounts of byproducts such as sugars [47]. Conversely, for the liquid residues, phenolic recovery was inherently linked to the thermal extraction that occurs during distillation itself relying solely on boiling to release water-soluble phenolics. These methodological differences likely influence both the quantity and the molecular profile of the bioactive components available for encapsulation, thereby impacting their stability and antioxidant performance during co-crystallization [48]. Likewise, the E/S parameter showed a strong positive correlation with AA (*p* = 0.000). An increase from 0.10 to 0.19 g/g was associated with a steep rise in AA, which then remained consistently high up to the maximum tested level of 0.70 g/g. This finding supports the hypothesis that greater concentration of liquid residue enhances phenolic retention, either through increased availability of active molecules or via delayed nucleation, allowing more efficient integration. The accumulation of phenolics, coupled with higher system density, may also strengthen interactions with sucrose, further promoting bioactive stabilization and protection. Conversely, in regions with simultaneously low Xs (50–55 °Brix) and E/S (<0.20 g/g), AA dropped significantly, with minimum values falling below 60%. These low values likely reflect insufficient phenolic entrapment or leakage during crystallization. The lack of a robust matrix and inadequate sucrose crystallinity appear to compromise the system’s ability to effectively protect phenolic components from degradation.

Moisture content (MC) is a critical quality parameter of encapsulated powders, as it directly influences their physicochemical stability, hygroscopicity, microbial safety, and overall shelf life. In co-crystallization processes, the system’s ability to release water and attain low residual moisture is closely related to its composition and rheological behavior during crystal network formation. In both solid and liquid residue systems, MC was strongly affected by the concentration of extract solids (Xs) and E/S ratio.

**Figure 10:**
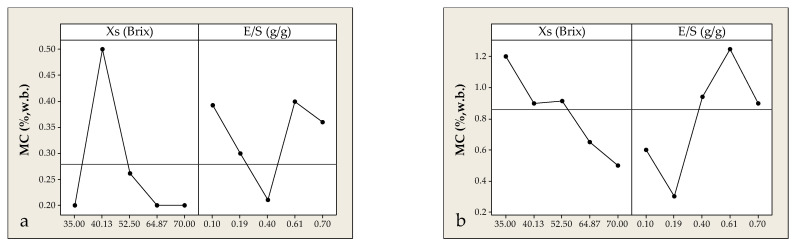
Main effects plot of solids concentration of extract (Xs, °Brix) and dry extract-to-sucrose ratio (E/S, g/g) on moisture content (MC, % w.b.) of co-crystallized powders produced using (**a**) solid and (**b**) liquid by-products from lavender distillation.

For solid residue powders, MC peaked (~0.50%) at Xs = 40 °Brix, then dropped sharply below 0.20% as Xs increased beyond 60 °Brix—indicating that supersaturation and higher viscosity promote rapid crystallization and efficient water removal. Similarly, E/S ratio had a more complex, nonlinear effect. The lowest values occurred at E/S = 0.40 g/g, while deviations in either direction led to slight moisture increases. These trends suggest an optimal compositional range that favors both dehydration and matrix uniformity. Moisture levels < 3% are typically targeted in co-crystallized powders. For instance, López-Córdoba and Navarro [49] encapsulated glucose in sucrose matrices and reported MC values ranging between 0.75 και 1.43%, while Bhandari and Hartel [14] observed values of 0.5 και 3.0% for glucose and fructose systems. Irigoiti et al. [36] reported moisture levels between 0.03 και 2.09% for propolis co-crystallization, and Federzoni et al. [50] noted 2.7% MC in paprika oleoresin encapsulated using supersaturated sucrose syrup. Figure 10a confirms these trends, showing steep MC reductions above 50 °Brix, consistent with the role of supersaturation in accelerating crystallization and enhancing water removal. Periodic moisture fluctuations were also observed with varying E/S ratios, with the lowest MC values (<0.25%) at intermediate E/S (0.20 g/g). In contrast, extreme E/S values (either low or high) led to higher moisture levels, potentially due to altered viscosity or disrupted crystal homogeneity. Comparable findings were reported by Ligarda-Samanez et al. [51], who showed that propolis and honey extract encapsulation yielded optimal MC values at intermediate E/S ratios. Similarly, Tzatsi and Goula [12] observed that higher extract solids concentrations led to lower intrinsic moisture and thus reduced final product moisture.

The moisture content behavior in powders derived from lavender distillation liquid residue followed a different pattern with important quantitative distinctions. As shown in Figure 10b, increasing Xs from 40 to 70 °Brix resulted in a sharp MC reduction from ~1.2 to ~0.4%, attributed to reduced water load and enhanced crystalline network formation under supersaturated conditions. The E/S ratio again showed a nonlinear effect, with the lowest MC (0.20%) at E/S = 0.19 g/g. Both lower and higher values led to moisture increases, peaking at ~1.2% for E/S = 0.61 g/g (Figure 10b). Regions where Xs > 50 °Brix were associated with steep moisture reductions, affirming the beneficial effect of supersaturation on crystal formation and water release. As with the solid-residue system, MC fluctuations were noted across the E/S range, with minimal moisture (<0.25%) found at intermediate ratios. Extreme E/S values likely disturb viscosity and network homogeneity, resulting in moisture retention. While both systems achieve desirable low moisture levels under optimized conditions, powder derived from liquid distillation residues tend to exhibit slightly higher initial MC compared to those from solid residues. The E/S ratio exerts a consistent parabolic influence, with optimal MC observed at intermediate values in both cases. These findings reinforce the need for careful parameter optimization, particularly of Xs and E/S, to ensure crystalline stability and minimal residual moisture in co-crystallized bioactive powders.

Solubility, assessed through dissolution time (S), is a key technological attribute for application requiring rapid hydration and reconstitution. In co-crystallized powders, dissolution kinetics depend strongly on matrix structure, which is in turn shaped by formulation and crystallization conditions. The maximum S (~90 s) was observed at Xs = 40 °Brix, followed by a marked decrease (~54 s) at Xs ≥ 52.5 °Brix. This trend suggests that intermediate Xs levels lead to denser, less porous matrices that slow water penetration, whereas higher Xs values may reduce sucrose content and loosen structure, facilitating faster solubilization. Shortest dissolution times (~54 s) were recorded at E/S = 0.10 and 0.40 g/g, while a peak (~72 s) occurred at E/S = 0.61 g/g, likely due to delayed crystallization and tighter molecular packing. Two solubility-limiting zones are observed: (i) Xs = 40 °Brix, E/S = 0.61 g/g and (ii) Xs > 65 °Brix, E/S < 0.20 g/g, corresponding to compact or heterogeneous matrices. Conversely, optimal solubility (S < 60 s) occurred in the Xs range of 50–60 °Brix, and E/S ≈ 0.20–0.40 g/g, likely due to the formation of more open, porous crystal networks. Literature comparisons reinforce these findings. Solubility values between 41 and 57 s were reported for similar encapsulated systems involving propolis, carrot, ginger oleoresin, and peppermint [36,52,53,54]. In all cases, phenolic content, particle morphology, and encapsulation structure were key solubility drivers.

**Figure 11:**
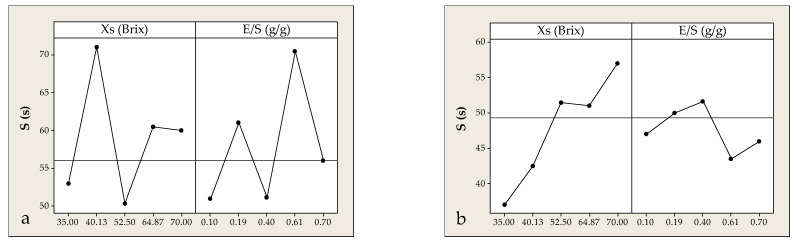
Main effects plot of solids concentration of extract (Xs, °Brix) and dry extract-to-sucrose ratio (E/S, g/g) on solubility (S, s) of co-crystallized powders produced using (**a**) solid and (**b**) liquid by-products from lavender distillation.

Dissolution behavior in powders derived from lavender liquid residues showed distinct dynamics. As shown in Figure 11b, dissolution time increased from ~44 s to ~56 s as Xs rose from 40 to 70 °Brix. This deceleration at high Brix is likely due to the formation of compact, less porous matrices. Intermediate concentration (Xs = 52.5 °Brix) allowed faster water penetration and structure breakdown. The E/S ratio again showed a nonlinear response. The lowest S (~45 s) was recorded at E/S = 0.61 g/g, with a maximum (~55 s) at E/S = 0.70 g/g, indicating that excessive phenolic load may hinder crystallization and lead to dense, slower-rehydrating structures. The present work also revealed complex interactions: highest S values (>56 s) occurred at Xs ≈ 35–40 °Brix and E/S ≈ 0.35–0.50 g/g. These conditions likely promote porous sucrose lattice formation, enhancing water diffusion and reconstitution. It is important to highlight that the extraction of bioactive components differed fundamentally between the two lavender distillation residues. For solid residues, microwave-assisted extraction with ethanol was employed—a method well-documented for enhancing both phenolic recovery and the co-extraction of sugars from matrices such as lavender stems. In contrast, phenolic recovery from liquid residues relied solely on thermal extraction via direct boiling during distillation, which is considerably less effective in extracting sugars or high-molecular-weight compounds. These methodological discrepancies in extract composition likely contributed to the structural density, solubility, and functional performance of the resulting powders. Although both residue systems exhibited similar nonlinear solubility responses, powders from solid residues reached higher maximum S values (~72 s), suggesting greater susceptibility to densification under certain conditions. In contrast, the liquid system exhibited shorter overall dissolution times but also greater sensitivity to E/S fluctuations. Researchers such as Sarabandi et al. [10] confirm that increased phenolic content may statistically reduce solubility due to changes in particle size, shape, and microstructure, aligning with trends observed in this work.

**Figure 12:**
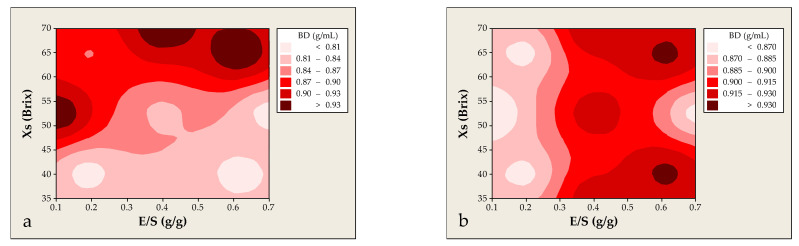
Effect of solids concentration of extract (Xs, °Brix) and dry extract-to-sucrose ratio (E/S, g/g) on bulk density (BD, g/mL) of co-crystallized powders produced using (**a**) solid and (**b**) liquid by-products from lavender distillation.

Bulk density (BD) is a key physicochemical property of encapsulated dry powders, influencing flowability. In this study, BD was assessed as a function of the Xs and E/S for both solid and liquid distillation residue systems. For powders derived from solid residues, BD increased steadily from ~0.83 to ~0.95 g/mL as Xs rose from 35 to 70 °Brix, indicating denser and less porous structures at higher solid loadings. This condition limited air entrapment during crystallization and enhanced structural density. Additionally, the elevated phenolic content contributed to matrix cohesion, facilitating closer packing of particles and increasing mass per volume unit. These findings are consistent with previous studies reporting similar BD values for co-crystallized powders from botanical sources such as yerba mate [15], marjoram [10], and aronia [12]. Maximum BD values were found at intermediate E/S values (0.10–0.19 g/g and 0.40–0.60 g/g), while minimum density (~0.83 g/mL) occurred at E/S = 0.70 g/g. This suggests that excessive extract loading may disrupt crystal network formation, increase porosity, and impair particle packing. Alterations in microstructure and surface morphology induced by extract–carrier interactions may also reduce compressibility and promote the formation of looser aggregates. Similar effects have been reported during the encapsulation of marjoram and propolis extracts [10,36]. Figure 12a highlights that BD values above 0.90 g/mL were typically observed at high Xs (>65 °Brix) and intermediate E/S ratios (0.10–0.20 and 0.40–0.60 g/g). Conversely, low BD values (<0.85 g/mL) were associated with low Xs (<50 °Brix) and high E/S ratios (>0.5 g/g), where weaker structural development likely resulted in porous and less cohesive particles.

For the powder obtained from liquid distillation residues, the overall BD range was similar, varying from <0.86 to >0.92 g/mL. However, the response patterns exhibited key differences. Increasing Xs from 35 to 70 °Brix resulted in a pronounced increase in BD, reaching a peak of ~0.93 g/mL. This aligns with the behavior observed in solid residues, indicating that elevated dry matter content promotes denser structures with reduced air retention and improved particle organization. The effect of the E/S ratio in this system also followed a nonlinear trend but with a distinct optimum at E/S ≈ 0.61 g/g, where the maximum BD (~0.94 g/mL) was recorded. At higher extract loads (E/S = 0.70 g/g), BD dropped significantly (~0.86 g/mL), likely due to excessive phenolic content interfering with crystal formation, increasing porosity, or altering surface characteristics. Additionally, high E/S values may induce greater internal heterogeneity, resulting in reduced compressibility and irregular, loosely packed granules. As shown in Figure 12b, optimal BD values (>0.92 g/mL) were achieved in areas where Xs > 65 °Brix and E/S = 0.40 g/g, or where Xs < 55 °Brix and E/S ranged from 0.50 to 0.70 g/g. These regions likely promote the formation of well-structured and stable aggregates due to a balanced matrix composition. In contrast, low BD values (<0.86 g/mL) were observed in formulations with Xs = 50–55 °Brix and E/S > 0.10 g/g, conditions that may foster less cohesive, porous structures due to high extract content and insufficient carrier matrix. In comparative terms, both systems demonstrated a positive correlation between Xs and BD, reinforcing the critical role of solid content in enhancing powder compactness. However, the solid residue system showed a wider BD range and greater sensitivity to E/S variations, possibly due to its richer phenolic and sugar profile derived from microwave-assisted extraction in aqueous ethanol, which facilitates the co-extraction of polysaccharides.

Hygroscopicity (H) is a critical physicochemical parameter of encapsulated products, as it reflects their capacity to absorb moisture from the environment. This parameter plays a crucial role in product stability during storage, susceptibility to caking, and behavior under high relative humidity conditions. The extent of hygroscopicity is closely linked to the composition and microstructure of the encapsulated matrix. One of the key factors is the physical structure of sucrose; crystalline sucrose exhibits significantly lower H than its amorphous form, regardless of the environmental conditions [14,16]. According to Chezanoglou and Goula [8], phenolic compounds incorporated into co-crystallized matrices can modulate H through interactions with sucrose. For instance, co-crystallized phenolic extracts displayed lower H values compared to their pure counterparts.

In this study, H was investigated as a function of Xs and E/S ratio. H values were remarkably low, with H ranging from −0.7% to 1.0%, indicating either negative or near-zero moisture uptake. Interestingly, some negative values were observed, indicating moisture loss during storage—a phenomenon that suggests exceptional physicochemical stability and negligible hygroscopic behavior due to the formation of structurally stable, dense, and low-porosity particles with minimal available surface area for water vapor adsorption. For solid waste, the highest H value was observed at E/S = 0.19 g/g, while the lowest occurred at E/S = 0.40 g/g. This pattern may reflect differences in the distribution of hydrophilic groups between sucrose and phenolics, and the manner in which they influence the crystalline lattice and its interaction with atmospheric moisture. Sarabandi et al. [10] reported similar trends in marjoram extract co-crystallization, noting that increasing extract concentration correlated with greater H. This trend is likely due to the elevated content of low-molecular-weight sugars (e.g., glucose, fructose) in concentrated extracts, which tends to retain an amorphous structure and bind water molecules, thereby increasing the system’s H [55,56]. Furthermore, previous studies on phenolic extract co-crystallization have consistently shown that increasing extract addition to saturated sucrose solutions leads to higher H of the final powder [10,57]. The ability to achieve negative H values demonstrated the potential of the co-crystallized powders for application requiring high physicochemical stability, reduced caking, extended shelf life, and improved technological performance during storage and handling.

Powders derived from liquid distillation residues exhibited an even lower hygroscopic profile. This behavior was particularly evident at Xs > 65 °Brix and E/S = 0.40 g/g, where the system favored the formation of compact, low-porosity structures with minimal available surface from moisture adsorption. In terms of the Xs variable, the H value remained relatively stable up to 65 Brix, followed by a sharp decrease at higher concentrations. This trend likely reflects a transition to more compact, less porous matrices as a result of supersaturation, which restricts water–solid interactions. By comparison, the solid-residue system showed a slightly greater sensitivity to E/S extremes, leading to modest increases in hygroscopicity under suboptimal ratios. This behavior is possibly attributed to the higher content of amorphous sugars and low-molecular-weight solutes retained during ethanol-based extraction—a method that not only enhances phenolic recovery but also promotes the co-extraction of compounds (e.g., glucose, fructose) with inherently higher hygroscopic potential and a tendency to remain in amorphous forms.

Comparable low H values for co-crystallized powders have also been reported by other researchers. Deladino et al. [15], in the co-crystallization of lactic acid in sucrose matrix, observed H values ranging from 0.24 και 2.33%. Similarly, López-Córdoba et al. [57] and Bajaj and Singhal [58], investigating co-crystallized zinc sulfate and vitamin B12 in supersaturated sucrose syrups, reported maximum H values of 2.00 and 2.51%, respectively.

**Figure 13:**
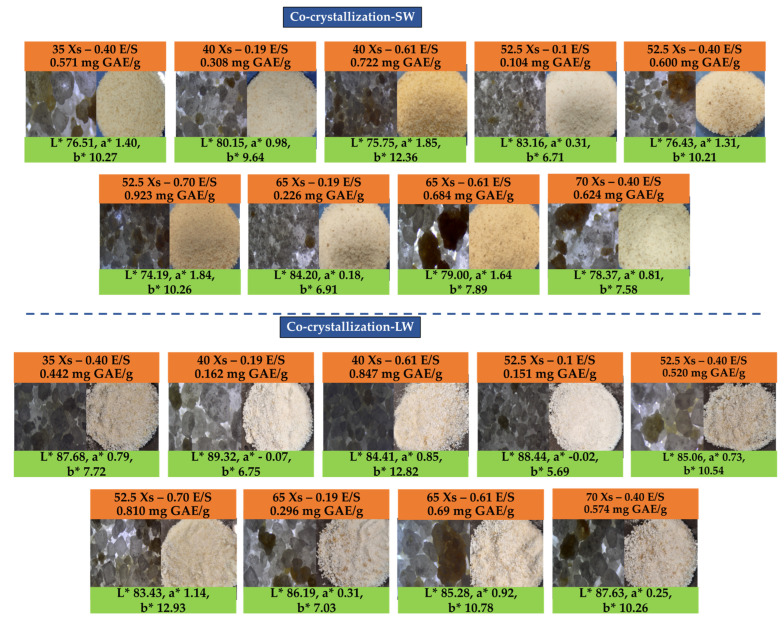
Morphology, color parameters, and phenolic content of encapsulated extracts of lavender distillation solid waste (SW) and liquid waste (LW) by the co-crystallization method.

Color is a critical quality attribute in encapsulated powder products, as it influences consumer perception of freshness, attractiveness, and differentiation in competitive markets. In this study, the colometric parameters L* (lightness), a* (red–green axis), and b* (yellow–blue axis), using the CIE-L*a*b* color space, were evaluated to determine the effects of Xs and the E/S ratio on the visual quality of the resulting co-crystallized powders. As shown in Figure 13, L* increased gradually with rising Xs up to 65 °Brix (L* ≈ 82), likely due to more uniform and transparent sucrose crystallization that enhances light scattering. However, at 70 °Brix, a slight decrease in L* was observed, possibly attributed to the increased presence of phenolic compounds containing endogenous chromophores. Similarly, L* declined steadily as the E/S ratio increased, from ~82 at E/S = 0.10 g/g to ~74 at E/S = 0.70 g/g, indicating a darkening effect related to higher pigment concentration or accumulation of dark-colored compounds. This trend agrees with previous findings by Sarabandi and Mohammadi [54], who observed a reduction in L* during co-crystallization of the phenolic mint extract at higher E/S ratios. Comparable results were also reported by López-Córdoba et al. [59] and Sarabandi et al. [10], who noted that higher amounts of phenolic extract in the formulation led to darker powders with lower L* values. Figure 13 provides visual confirmation of powder appearance under different encapsulation conditions, further validating the influence of phenolic content and crystallization dynamics on final product characteristics. Color parameter ranges reported by other studies using co-crystallized phenolic-rich extracts were consistent. Karangutkar and Ananthanarayan [17] observed L* values ranging from 49.35 to 62.10 for the B. rura extract. Ιrigoiti et al. [36] reported a broader range of 62.00–82.26, while Sarabandi and Mohammadi [54] recorded values between 61.77 and 63.74 for peppermint phenolics. Similarly, Sarabandi et al. [10], Khawas and Deka [56], and Tzatsi and Goula [12] reported values of 63.83–65.31, 67.75–70.86, and 63.58 ± 0.49, respectively, for marjoram, banana pulp/peel, and Aronia extracts. Conversely, López-Córdoba et al. [59] and Kaur et al. [52] reported lower L* values (55.3–55.9 and 30.3–36.3, respectively), emphasizing the influence of extract type and pigment content. Regarding the a* parameter values, increasing the E/S ratio up to 0.61 g/g enhanced redness, while a further increase to 0.70 g/g resulted in a decline in a*, suggesting pigment instability or incomplete incorporation into the crystalline matrix. The b* parameter decreased with increasing Xs, likely due to the emergence of darker pigments such as melanoidins. Conversely, increasing E/S from 0.10 to 0.70 g/g was associated with a progressive increase in b* (from 7.5 to 10.5), potentially reflecting the stabilization of heat-sensitive or yellowish phenolic compounds within the crystalline structure. Across the literature, co-crystallized powders generally exhibit positive a* values, indicating a reddish hue. Sarabandi et al. [10] and Sarabandi and Mohammadi [54] reported a* values in the range of 2.92–3.36 and 1.98–3.33, respectively, while Kaur et al. [52] observed values between 4.8 and 6.8 for carotenoid extracts. Irigoiti et al. [36] and Tzatsi and Goula [12] reported higher values (3.99–11.03 and 9.44 ± 0.1) for propolis and Aronia extract powders, respectively.

The chromatic behavior of co-crystallized powders derived from solid and liquid lavender distillation residues differed notably due to compositional and structural variations introduced during extraction and processing. Both systems showed color variations in response to Xs and E/S, but the magnitude and direction of these changes were distinct. In terms of lightness, powders from liquid distillation by-products consistently exhibited higher brightness values (L* = 83–89), particularly under low E/S (0.10–1.20 g/g) and Xs ≤ 45 °Brix. This suggests more homogeneous crystallization and a reduced colorant presence in the system. Conversely, solid-residue-derived powders demonstrated lower lightness (L* = ~74–82.5) and a stronger dependence on both E/S and Xs. The a* parameter also followed distinct trends. In the liquid residue system, a* values remained low but increased steadily with E/S, indicating moderate enhancement of red hues due to stabilized phenolic pigments. The solid-residue system, however, reached much higher a* values under simultaneous high E/S and Xs conditions, pointing to greater incorporation and possibly better stabilization of red pigments within the crystalline matrix.

These results align with prior findings for polyphenol encapsulation [10,12], suggesting that ethanol-extracted matrices are more chromatically intense. As for the b* parameter, the liquid-residue powders showed a clear and predictable increase in b* with E/S, reflecting an increased presence of yellowish or thermosensitive phenolics, whose stability is enhanced through the co-crystallization process. The highest b* values (>12) occurred at E/S > 0.60 g/g and Xs between 40 and 55 °Brix, pointing to the presence of yellow components retained from the distillation process. In contrast, the solid residue powders exhibited broader fluctuations, with b* ranging from ~7.5 to >12 depending on the interaction of high phenolic loads and sucrose concentration. The presence of more thermolabile chromophores and reducing sugars in the solid extracts—extracted via microwave-assisted extraction in ethanol—may have enhanced Maillard-derived yellowing. Overall, the liquid-residue system favored lighter, more neutral-colored powders with improved visual uniformity, while the solid-residue system produced darker, more saturated tones due to greater pigment and sugar co-retention. These differences emphasize the critical role of extraction method (boiling vs. ethanol microwaves) not only on the phenolic composition but also on the chromatic quality and visual appeal of the final encapsulated product.

## 4. Conclusions

This study highlights advantages and limitations of two encapsulation strategies—co-crystallization and ionic gelation—for stabilizing bioactive compounds derived from lavender distillation residues. Notably, the co-crystallization process demonstrated a markedly superior Ef (>150%) compared to ionic gelation (~40%), attributable to the unique ability of crystalline sucrose matrix to entrap active components within its developing lattice structure. Although both techniques preserved antioxidant capacity, co-crystallization provided greater functional stability across a wider spectrum of formulation parameters, underscoring its robustness and versatility. From a technological standpoint, the co-crystallized powders exhibited significantly lower residual moisture content (<0.5%) than the extruded beads (94.0–95.4%), positioning them as ideal candidate for applications requiring low water activity and extended shelf life. Moreover, the occurrence of negative hygroscopicity values in co-crystallized products serves as a strong indicator of exceptional physicochemical reliance against moisture uptake. Their dissolution time was also notably reduced (<60 s under optimal conditions), likely due to their porous structure and efficient dispersion of phenolics within the matrix, whereas this parameter was not applicable to the extruded systems. Additionally, co-crystallization enabled greater tunability in bulk density and offered refined control over the chromatic properties, with clear correlations to formulation variables such as phenolic content and E/S ratio. While ionic gelation may offer benefits in terms of morphological control—such as a consistent granule size—co-crystallization outperformed it in key parameters including encapsulation efficiency, hygroscopic stability, and functional adaptability.

In conclusion, a comparative evaluation of the two encapsulation techniques demonstrates distinct advantages and limitations inherent to each method. Ionic gelation proved effective for forming uniform, spherical microcapsules with high encapsulation efficiency and moderate moisture content, making it suitable for applications requiring controlled release or dispersion in aqueous food matrices. However, it involved additional processing steps and the use of calcium chloride, which may limit its scalability in certain production environments. On the other hand, co-crystallization offered a simple and low-cost approach, yielding dry, sugar-based microstructures with good flowability and long shelf-life potential. Despite its convenience, the method showed relatively lower encapsulation efficiency and limited protection of phenolics under simulated storage conditions. These findings suggest that while ionic gelation may be more appropriate for functional food ingredients requiring bioactive stability and targeted delivery, co-crystallization can serve as a practical alternative for solid formulations or applications where ease of handling and stability are prioritized.

The novelty of this work lies in its dual focus on both valorization of agro-industrial by-products and the advancement of gentle, scalable encapsulation techniques tailored for heat-sensitive phytochemicals. By applying co-crystallization to underutilized aromatic plant waste streams, this study not only contributes to circular bioeconomy principles but also pioneers the development of high-performance, low-moisture delivery systems for bioactives. Our findings extend the current body of research by providing a systematic, side-by-side evaluation of ionic gelation and co-crystallization under identical bioactive payloads—bridging a notable gap in encapsulation research. Collectively, this research sets a strong foundation for sustainable innovation in plant-based ingredient stabilization and highlights co-crystallization as a promising tool for the future of green food and pharmaceutical technologies.

Nonetheless, certain limitations must be acknowledged. Although sodium alginate and sucrose were selected in this study due to their low cost, food-grade status, and industrial applicability, it is acknowledged that alternative encapsulating matrices may enhance encapsulation efficiency and compound stability. Future work will explore such materials to optimize performance across different application scenarios. Long-term stability studies under various environmental conditions are needed to validate the performance of the encapsulated products over time. Future research should aim to optimize encapsulation conditions for other classes of bioactives and explore the integration of these techniques into industrial-scale applications to fully realize their commercial and environmental potential. Finally, both techniques would benefit from comprehensive release kinetics modeling and scale-up validation to support their broader adoption in functional food and pharmaceutical formulations.

## Figures and Tables

**Table 1:** Experimental values of the independent variables of each encapsulation method.

Independent Variable (Symbol)	Unit	Levels				
Encapsulation by Ionic Gelation						
Concentration of sodium alginate solution (Calg)	% w/w	2		2.5		3
Concentration of extract (Ce)	% w/w	1		7.5		15
Encapsulation by Co-Crystallization						
Solids concentration of extract (Xs)	°Brix	35	40	52.5	65	70
Dry extract to sucrose ratio (E/S)	g/g	0.1	0.19	0.4	0.61	0.7

## Data Availability

The datasets used and/or analyzed during the current study are available from the corresponding author on reasonable request.

## Abbreviations

The following abbreviations are used in this manuscript:

Ef	Encapsulation efficiency
AA	Antioxidant activity
MC	Moisture content
EO	Essential oil
SW	Solid waste
LW	Liquid waste or leachate
UAE	Ultrasound-assisted extraction
MAE	Microwave-assisted extraction
GAE	Gallic acid equivalents
d.w.	Dry weight
Calg	Concentration of sodium alginate solution
Ce	Concentration of extract
CaCl_2_	Calcium chloride
Xs	Solids concentration of extract
E/S	Dry extract to sucrose ratio
S	Solubility-Wetting time
L*	Lightness
BD	Bulk density
H	Hygroscopicity
HNO_3_	Nitric acid
DPPH	1,1-diphenyl-2-picrylhydrayl
I	Inhibition
